# Mobilizing primary biodiversity records in environmental assessments in Spain

**DOI:** 10.3897/BDJ.13.e142302

**Published:** 2025-03-25

**Authors:** Maite Telletxea, Rafael Miranda, Arturo H. Ariño, David Galicia

**Affiliations:** 1 Universidad de Navarra, Instituto de Biodiversidad y Medioambiente BIOMA, Irunlarrea 1, 31008, Pamplona, Spain Universidad de Navarra, Instituto de Biodiversidad y Medioambiente BIOMA, Irunlarrea 1, 31008 Pamplona Spain; 2 Universidad de Navarra, Facultad de Ciencias, Departamento de Biología Ambiental, Biodiversity Data Analytics and Environmental Quality (BEQ), Irunlarrea 1, 31008, Pamplona, Spain Universidad de Navarra, Facultad de Ciencias, Departamento de Biología Ambiental, Biodiversity Data Analytics and Environmental Quality (BEQ), Irunlarrea 1, 31008 Pamplona Spain

**Keywords:** environmental assessment, records of decision, dark data, primary biodiversity records, GBIF

## Abstract

**Background:**

Environmental Assessment is an essential tool for minimising the environmental impact of human development, generating huge amounts of biodiversity data. However, much of this information, also in Spain, remains inaccessible after being partially included in Records of Decision (RODs). As a result, these dark data remain under-utilised, limiting their potential to provide information for conservation efforts and decision-making processes.

**New information:**

This dataset compiles 4,630 species records derived from RODs published in the Spanish Official State Gazette between 2013 and 2023, focusing on catalogued species listed in the Spanish Catalogue of Threatened Species and the List of Wild Species under Special Protection Regime. The data were collected using automated text-mining techniques and manually curated to correct errors and classify records as primary biodiversity records (PBRs), absences or literature-based occurrences. A total of 1,290 PBRs, 170 absences and 3,391 literature-based records were identified. PBRs were georeferenced and standardised according to the Darwin Core Standard. This dataset offers valuable insights into the presence and distribution of 31 non-Chiroptera species and 28 Chiroptera species, including 12 endangered (EN), 31 vulnerable (VU) and 16 listed species. The publication of these data in a FAIR (Findable, Accessible, Interoperable and Reusable) format via the Global Biodiversity Information Facility enhances their accessibility for future conservation planning and decision-making processes.

## Introduction

Environmental Assessment (EA) is the leading technical and administrative procedure for controlling human development impacts on biodiversity in many countries, including Spain (Weaver et al. 2008,Dias et al. 2022). This procedure was first introduced into Spanish regulations in 1986 for the Environmental Impact Assessment (EIA) mandated prospective projects and, later, in 2006 for the Strategic Environmental Assessment (SEA) for plans and programmes (Ley 21/2013, de 9 de diciembre, de evaluación ambiental 2013) to minimise the negative environmental impacts of human development (Dias et al. 2022).

Environmental Assessments generate many primary biodiversity data or records (PBRs), a potentially valuable source of information to assist management and conservation plans and guide decision-making (Weaver et al. 2008). Of all details in the EA procedure, only the Records of Decision (RODs) become permanently published and remain available to the public because their issuance is mandatory to continue the EA procedure (Ley 21/2013, de 9 de diciembre, de evaluación ambiental 2013). These mandatory and determining public pronouncements, such as environmental impact reports (Informe de Impacto Ambiental, IIA), environmental impact statements (Declaración de Impacto Ambiental, DIA), strategic environmental reports (Informe Ambiental Estratégico, IAE) and strategic environmental statements (Declaración Ambiental Estratégica, DAE), are synthesis documents that do not provide accessible, standardised or actionable data and commonly do not even include all the biodiversity data generated as part of EA (Ley 21/2013, de 9 de diciembre, de evaluación ambiental 2013).

Therefore, EA-related biodiversity data are generally unavailable for reuse to provide information for future studies, scientific research, environmental planning and decision-making after the EA is completed (King et al. 2012, Stall et al. 2019), remaining as "dark" data (Heidorn 2008). If they can be made Findable, Accessible, Interoperable and Reusable (FAIR, Wilkinson et al. 2016), they can become a powerful tool for better decision-making and conservation planning, especially when dealing with protected or threatened species. In the case of Spain, the legal instruments for the conservation of wild species are the List of Wild Species under Special Protection Regime (Listado de Especies Silvestres en Régimen de Protección Especial, LESRPE) and the Spanish Catalogue of Threatened Species (Catálogo Español de Especies Amenazadas, CEEA).

The inclusion of a species (or population) within these catalogues mandates their periodic assessment, which would be more accurate if there is access to the largest and best existing evidence, including the EA-related data. One of the most recognised web infrastructures established to make the world’s biodiversity data freely, universally and digitally available to anyone is the Global Biodiversity Information Facility (GBIF) (Edwards 2001, Curry and Humphries 2007). Though incomplete, data mobilised through GBIF could provide both a first approach to what is readily available and a stable repository for the information in the above-mentioned instruments.

## General description

### Purpose

This dataset aims to surface as FAIR the dark data generated during EA and stuck in RODs. Publishing these data in a publicly accessible facility such as GBIF provides an opportunity for these data with great potential to be reused for future conservation decisions based on the best available evidence.

### Additional information

A database of 5,338 species records was completed during the RODs' revision (Suppl. material 1). These data included 487 records corresponding to automatic detection errors, 248 species records not automatically detected and 4,510 biodiversity records corresponding to correctly detected species, which rose to 4,603 records due to the occasional presence of many records of one species in the same ROD. Amongst the actual species records, a total of 1,290 corresponded to PBRs, a total of 170 corresponded to absences and 3,391 corresponded to bibliographic data. We found a total of 37 duplicated PBRs.

Excluding those duplicates, the 1,263 PBRs of the species of interest were submitted to GBIF.

## Sampling methods

### Sampling description

We collected EA-related reports by searching the Official State Gazette (BOE, https://www.boe.es/, accessed on 21 February 2023) for the terms “evaluación ambiental” (Spanish for "environmental assessment[s]"). We selected those documents appearing in the "Other provisions" database, where RODs were found. We created a workflow to extract their full content as character strings using Octoparse 8.5.3, a Windows-based data scraper (Octoparse 2024). This procedure let us detect information or patterns in the RODs using regular expressions or R packages for basic string processing and manipulation, such as *stringr*.

We automatically searched for occurrence data in each document that matched the species of interest's scientific name, common names or synonyms considered in the LESRPE and the CEEA using R 4.1.1 (R Core Team 2022). We created a preliminary database gathering the scientific, common name or synonym of each recorded species and the ROD where they were cited. We interpreted all scientific name strings into unified taxon concepts (hereinafter "species") and eliminated duplicated records.

**Figure 1.**
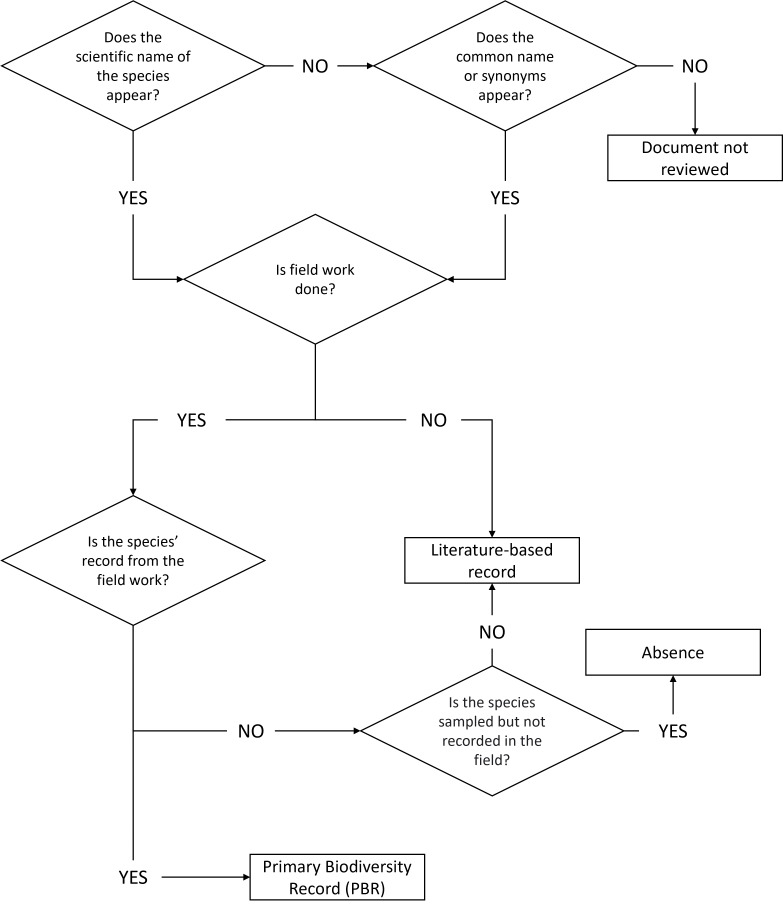
Decision diagram followed for categorising the species data recovered from RODs into three types: Primary Biodiversity Records (PBRs), literature-based citations and absence.

We manually categorised these data into three types based on the origin and the information they provided (Fig. 1). During the manual revision, we added those species records that were not automatically detected.

We classified occurrence data as PBRs when it appeared explicitly written in the ROD that fieldwork had been done in the study and the species record originated in that fieldwork. If a species was sampled for, but not recorded during the fieldwork and the plan's promoter explicitly stated it, the occurrence data was categorised as an absence. Otherwise, there being no formal evidence of the species having been recorded or looked for during the fieldwork, we recorded the alleged occurrence as literature-based.

The PBR's fieldwork timespan, if specified, was annotated in the ‘*verbatimEventDate*’ column. Based on this, the ‘*eventDate*’ field was filled with the single year or the multi-year period stated in the original document. Otherwise, the ROD publication year was used for the record.

PBRs were georeferenced a posteriori using Google Maps (https://www.google.com/maps). The record location was set to the coordinates of a point at the centre of the study area unless more specific information about the species' location was available. Uncertainty was inherent in generating coordinates for records and, generally, it was set to the radius of the smallest circle encompassing the whole study area from that central point following best practice guidelines (Chapman and Wieczorek 2020, Marcer et al. 2020, Marcer et al. 2022). All PBRs were georeferenced as decimal degrees to 1/1000th of a degree.

The species PBRs retrieved from the RODs were incorporated to the MZNA database (Zootron v.4.5, Ariño (1991)) and were made fully compliant with the DwC Standard (Darwin Core Maintenance Group 2023) for their publication in GBIF, compiling a database with 38 fields.

### Quality control

The performance of the automatic biodiversity data detection on RODs was estimated by determining the number of records correctly detected, the number of records that could not be detected and the number of detection errors. This information was gathered in the ‘*occurrenceRemarks*’ field. Taking as reference the typical confusion matrix of classification models, “precision” and “recall” were calculated (Kohavi and Provost 1998, Fahmy Amin 2022).

In a typical classification model, the precision is the ratio of the number of samples correctly classified as positive (True Positive, TP) to the number of all samples (TP and False Positive, FP) classified as positive (Kohavi and Provost 1998, Fahmy Amin 2022). The “precision” of the automatic detection was estimated as the ratio of actual biodiversity data detected to the number of all detections. In a typical classification model, recall is the ratio of the number of TP to the number of all actual positive samples (TP and False Negative, FN) (Kohavi and Provost 1998, Fahmy Amin 2022). The “recall” of the automatic detection was estimated as the proportion of the total biodiversity records that were automatically detected. The performance of the automatic detection of biodiversity data was satisfactory, as both “precision” (0.937) and “recall” (0.948) showed high values. The FP, including species names that constitute the name of organisations (i.e. “Fundación oso pardo”) or incorrectly addressed species (i.e. “Lince Ibérico (*Lynxlynx*)”) compromised the “precision” of the detection and the misspelled or incomplete species names (i.e. “Alimoche (*Neophoronpernopterus*)” or those not included in the search (i.e. “águila-azor perdicera”) compromised the “recall” of the detection.

Georeferencing uncertainty was determined according to Marcer et al. (2020).

## Geographic coverage

### Description

**Figure 2.**
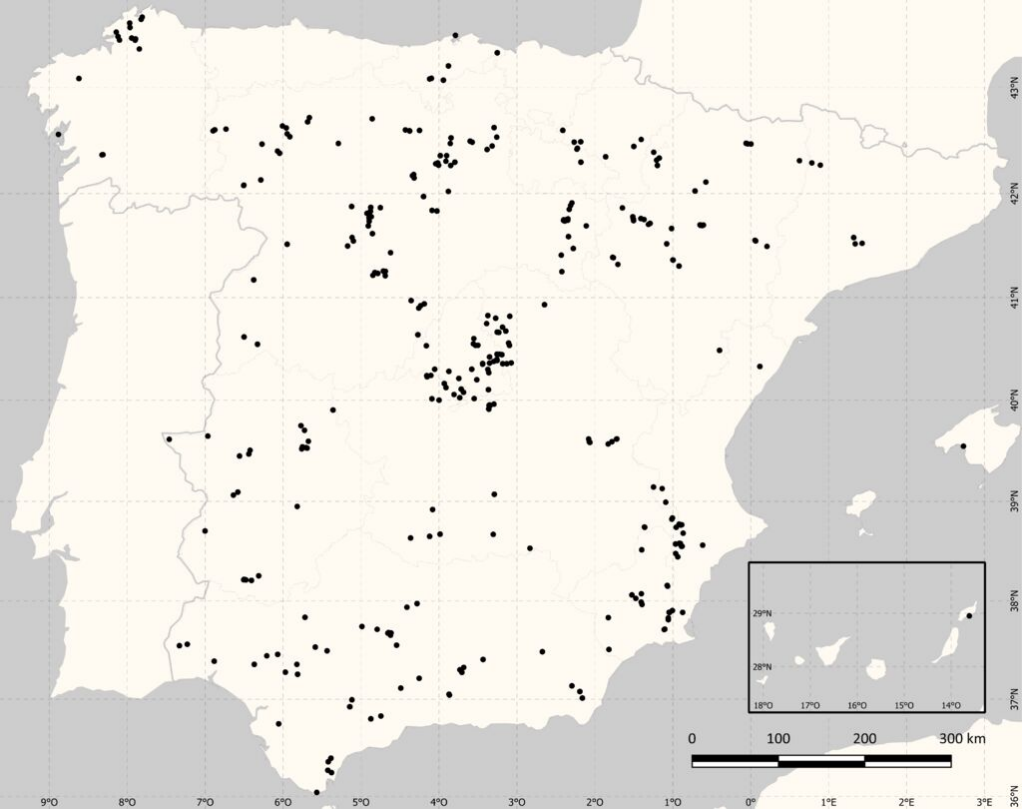
Spatial coverage of the fieldworks conducted during EA.

Spain (Fig. 2). The dataset comprises PBRs on EA fieldwork locations with an average uncertainty of 5,877.5 m, a minimum uncertainty of 500 m and a maximum of 26,000 m.

### Coordinates

28.951 and 43.659 Latitude; -13.61 and 2.729 Longitude.

## Taxonomic coverage

### Description

**Figure 3.**
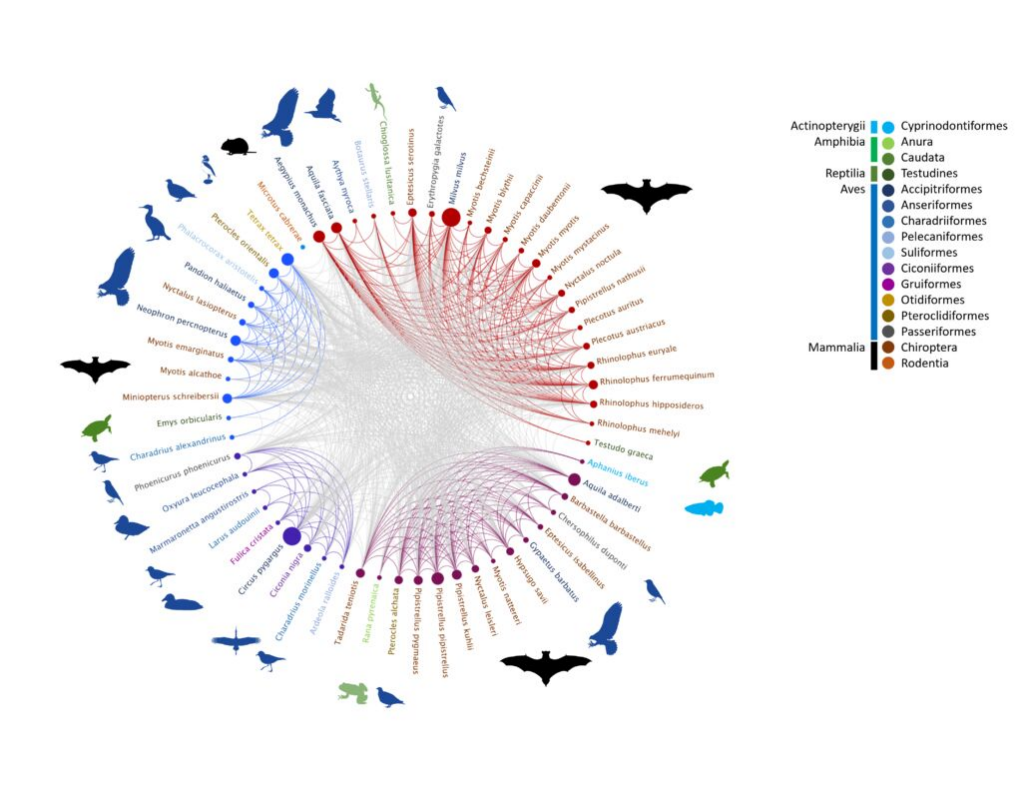
Circular graph of the 59 species (nodes) with PBRs in RODs and the relationship (edges) between them, meaning their coincidence in the same ROD.

The species of interest were the 20 Chiroptera species included in the LESRPE, the 13 Chiroptera species included in the CEEA as endangered (EN, one species) and vulnerable (VU, 12 species) and the 106 non-Chiroptera species included in the CEEA as EN (49 species) and VU (57 species). The dataset comprise the PBRs detected in the RODs belonging to 31 non-Chiroptera threatened species listed in the CEEA (11 EN species and 20 VU species) and 28 Chiroptera species (1 EN species, 11 VU species and 16 LESRPE species) present in Spain (Fig. 3).

### Taxa included

**Table:** 

Rank	Scientific Name	
species	*Aegypiusmonachus* (Linnaeus, 1766)	
species	*Aphaniusiberus* (Valenciennes, 1846)	
species	*Aquilaadalberti* C.L. Brehm, 1861	
species	*Aquilafasciata* Vieillot, 1822	
species	*Ardeolaralloides* (Scopoli, 1769)	
species	*Aythyanyroca* (Güldenstädt, 1770)	
species	*Barbastellabarbastellus* (Schreber, 1774)	
species	*Botaurusstellaris* (Linnaeus, 1758)	
species	*Charadriusalexandrinus* Linnaeus, 1758	
species	*Charadriusmorinellus* Linnaeus, 1758	
species	*Chersophilusduponti* (Vieillot, 1824)	
species	*Chioglossalusitanica* Bocage, 1864	
species	*Ciconianigra* (Linnaeus, 1758)	
species	*Circuspygargus* (Linnaeus, 1758)	
species	*Emysorbicularis* (Linnaeus, 1758)	
species	*Eptesicusisabellinus* (Temminck, 1840)	
species	*Eptesicusserotinus* (Schreber, 1774)	
species	*Erythropygiagalactotes* (Temminck, 1820)	
species	*Fulicacristata* Gmelin, 1789	
species	*Gypaetusbarbatus* (Linnaeus, 1758)	
species	*Hypsugosavii* (Bonaparte, 1837)	
species	*Larusaudouinii* Payraudeau, 1826	
species	*Marmaronettaangustirostris* (Ménétries, 1832)	
species	*Microtuscabrerae* Thomas, 1906	
species	*Milvusmilvus* (Linnaeus, 1758)	
species	*Miniopterusschreibersii* (Kuhl, 1817)	
species	*Myotisalcathoe* von Helversen & Heller, 2001	
species	*Myotisbechsteinii* (Kuhl, 1817)	
species	*Myotisblythii* (Tomes, 1857)	
species	*Myotiscapaccinii* (Bonaparte, 1837)	
species	*Myotisdaubentonii* (Kuhl, 1817)	
species	*Myotisemarginatus* (E.Geoffroy, 1806)	
species	*Myotismyotis* (Borkhausen, 1797)	
species	*Myotismystacinus* (Kuhl, 1817)	
species	*Myotisnattereri* (Kuhl, 1817)	
species	*Neophronpercnopterus* (Linnaeus, 1758)	
species	*Nyctaluslasiopterus* (Schreber, 1780)	
species	*Nyctalusleisleri* (Kuhl, 1817)	
species	*Nyctalusnoctula* (Schreber, 1774)	
species	*Oxyuraleucocephala* (Scopoli, 1769)	
species	*Pandionhaliaetus* (Linnaeus, 1758)	
species	*Phalacrocoraxaristotelis* (Linnaeus, 1761)	
species	*Phoenicurusphoenicurus* (Linnaeus, 1758)	
species	*Pipistrelluskuhlii* (Kuhl, 1817)	
species	*Pipistrellusnathusii* (Keyserling & Blasius, 1839)	
species	*Pipistrelluspipistrellus* (Schreber, 1774)	
species	*Pipistrelluspygmaeus* (Leach, 1825)	
species	*Plecotusauritus* (Linnaeus, 1758)	
species	*Plecotusaustriacus* (J.Fischer, 1829)	
species	*Pteroclesalchata* (Linnaeus, 1766)	
species	*Pteroclesorientalis* (Linnaeus, 1758)	
species	*Ranapyrenaica* Serra-Cobo, 1993	
species	*Rhinolophuseuryale* Blasius, 1853	
species	*Rhinolophusferrumequinum* (Schreber, 1774)	
species	*Rhinolophushipposideros* (Bechstein, 1800)	
species	*Rhinolophusmehelyi* Matschie, 1901	
species	*Tadaridateniotis* (Rafinesque, 1814)	
species	*Testudograeca* Linnaeus, 1758	
species	*Tetraxtetrax* (Linnaeus, 1758)	

## Temporal coverage

### Notes

**Figure 4.**
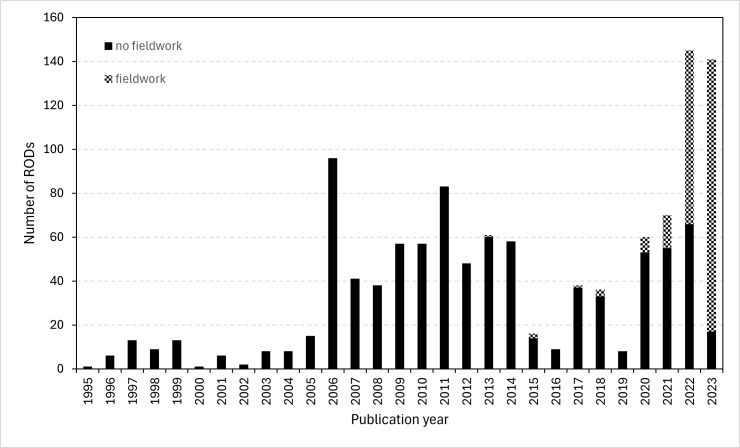
Number of RODs conducting or not fieldwork each year from May 1995 to February 2023.

Records of Decision published in the BOE between 1994 and February 2023 were reviewed. The automatic revision detected species records in RODs published between May 1995 and February 2023 (Fig. 4). The dataset comprised PBRs generated during EA-related fieldwork and included in RODs published between 2013 and 2023.

We observed an increase in the number of studies carrying out fieldwork amongst the documents that cite species, particularly in the last four years. In 2020 and 2021, only 11.7% (7 out of 60) and 21.4% (15 out of 70) of the published RODs, respectively, provided PBRs produced during fieldwork. In 2022, the percentage rose to 54.5% (79 out of 145) of the pronouncements providing new PBRs produced during fieldwork. Finally, in 2023, the percentage rose to 87.9%, so 124 RODs out of 141 provided new PBRs generated during fieldwork (Fig. 4, Suppl. material 1).

## Usage licence

### Usage licence

Other

### IP rights notes

Creative Commons Attribution Non-Commercial (CC-BY-NC 4.0)

## Data resources

### Data package title

Literature records in MZNA-LIT: primary biodiversity records in environmental assessments in Spain

### Resource link

https://doi.org/10.15470/bvznpy

### Alternative identifiers

https://www.gbif.org/dataset/3d6fbb6d-8699-4f93-9adc-c1fd6bc03f4e

### Number of data sets

3

### Data set 1.

#### Data set name

Occurrence

#### Data format

Tab-separated values, Darwin Core Standard

#### Download URL

https://doi.org/10.15470/bvznpy

#### Description

Thirty-eight fields were used to map the 1,263 PBRs of the species catalogued in the LESRPE and the CEEA named on RODs. Column description of type, modified, language, licence, references, institutionID, datasetID, institutionCode, collectionCode, basisOfRecord, occurrenceID, catalogNumber, occurrenceStatus, eventDate, continent, country, countryCode, locality, decimalLatitude, decimalLongitude, geodeticDatum, coordinateUncertaintyInMeters, scientificName, kingdom, phylum, order, family, genus, specificEpithet, taxonRank and vernacularName are based on the Darwin Core (DwC) Standard (Darwin Core Maintenance Group 2023).

**Data set 1.** 

Column label	Column description
id	Character. Unique identification for the record.
type	Character. The nature or genre of the resource.
modified	Numeric (integer). The most recent date-time on which the resource was changed.
language	Character. A language of the set of data.
licence	Character. A legal document giving official permission to do something with the resource.
references	Character. An identifier for the set of information associated with the same ROD.
institutionID	Character. An identifier for the institution having custody of the record.
datasetID	Character. A unique identifier for the set of data.
institutionCode	Character. The name (or acronym) in use by the institution having custody of the record.
collectionCode	Character. The name, acronym, coden or initialism identifying the collection or dataset from which the record was derived.
basisOfRecord	Character. The specific nature of the data record. Controlled vocabulary: MaterialCitation.
dynamicProperties	Character. The species category on the CEEA or LESRPE. Controlled vocabulary: {"ceeaCategory":"EN"}, {"ceeaCategory":"VU"} or {"ceeaCategory":"LESRPE"}.
occurrenceID	Character. A unique identifier for the species PBR found in the ROD.
catalogNumber	Number (integer). A unique identifier for the record within the collection.
individualCount	Numeric (integer). The number of organisms indicated on the ROD, if applied.
occurrenceStatus	Character. A statement about the presence or absence of the species in a specific location. Controlled vocabulary: PRESENT.
occurrenceRemarks	Character. Data type depending on the automatic detection in the ROD of the scientific name, vernacular name or synonyms of the species. Controlled vocabulary: detected, not detected or detection error.
eventDate	Character. The date-time or interval during the occurrence data was generated. It is based on the verbatimEventDate or the ROD publication year.
verbatimEventDate	Character. The period in which the EA fieldwork was conducted.
continent	Character. The name of the continent in which the species was sampled.
country	Character. The name of the country in which the species was sampled.
countryCode	Character. The standard code for the country in which the species was sampled.
locality	Character. Municipality where the occurrence data was recorded.
decimalLatitude	Numeric (double). The geographic latitude (in decimal degrees, using the spatial reference system given in geodeticDatum) of an occurrence record.
decimalLongitude	Numeric (double). The geographic longitude (in decimal degrees, using the spatial reference system given in geodeticDatum) of an occurrence record.
geodeticDatum	Character. The ellipsoid, geodetic datum or spatial reference system (SRS) upon which the geographic coordinates given in decimalLatitude and decimalLongitude are based.
coordinateUncertaintyInMeters	Number (integer). The horizontal distance (in metres) from the given coordinates describing the radius of the smallest circle encompassing the whole study area.
verbatimIdentification	Character. The scientific, common name or synonym of the species found in the ROD.
scientificName	Character. The full accepted scientific name, with authorship and date information, of the species found in the ROD.
kingdom	Character. The full scientific name of the kingdom in which the species found in the ROD is classified.
phylum	Character. The full scientific name of the phylum in which the species found in the ROD is classified.
class	Character. The full scientific name of the class in which the species found in the ROD is classified.
order	Character. The full scientific name of the order in which the species found in the ROD is classified.
family	Character. The full scientific name of the family in which the species found in the ROD is classified.
genus	Character. The full scientific name of the genus in which the species found in the ROD is classified.
specificEpithet	Character. The name of the species epithet of the scientificName.
taxonRank	Character. The taxonomic rank of the most specific name.
vernacularName	Character. The common name of the species found in the ROD.

### Data set 2.

#### Data set name

Reference

#### Data format

Tab-separated values, Darwin Core Standard

#### Download URL

https://doi.org/10.15470/bvznpy

#### Description

The fields listed in the Literature References Extension of the DwC used to support the bibliographic information of the Records of Decision. The definitions of identifier, bibliographicCitation, creator and rights are based on the Dublin Core Metadata Element Set (Dublin Core Metadata Initiative 2020). The definitions of title, date, subject and language are based on the GBIF DwC Extension for literature references (GBIF 2015). The definition of datasetID is based on the DwC Standard (Darwin Core Maintenance Group 2023).

**Data set 2.** 

Column label	Column description
id	Character. Unique identification for the record.
identifier	Character. An unambiguous reference (URL) to the Record of Decision.
bibliographicCitation	Character. A bibliographic reference for the Record of Decision.
title	Character. Title of the referenced Record of Decision.
creator	Character. An entity primarily responsible for making or publishing the Record of Decision.
date	Character. Date of publication of the Record of Decision.
source	Character. The reference to the Spanish Official State Gazette in which the Record of Decision is published.
subject	Character. Semi-colon-separated list of keywords.
language	Character. ISO 639-1 language code indicating the language of the Record of Decision.
rights	Character. Information about rights held in and over the Record of Decision.
datasetID	Character. A unique identifier for the set of data.

### Data set 3.

#### Data set name

Suppl. material 1: Species records recovered from RODs

#### Data format

Tab-separated values

#### Description

This is a database provided in the Supplementary material which comprises all the species records recovered from RODs, including Primary Biodiversity Records (PBRs), literature-based citations and absences. The database has 25 non-standardised fields.

(*) Mandatory fields only for PBR.

**Data set 3.** 

Column label	Column description
id	Character. Unique identification for the record.
detection	Character. Corresponds to the automatic detection of the species names. Controlled vocabulary: detected, not detected or detection error.
BOEdocument	Character. Document reference on the BOE.
kingdom	Character. The full scientific name of the kingdom in which the species found in the RODs is classified.
phylum	Character. The full scientific name of the phylum in which the species found in the RODs is classified.
class	Character. The full scientific name of the class in which the species found in the RODs is classified.
order	Character. The full scientific name of the order in which the species found in the RODs is classified.
family	Character. The full scientific name of the family in which the species found in the RODs is classified.
genus	Character. The full scientific name of the genus in which the species found in the RODs is classified.
species	Character. The full scientific name of the species in which the species found in the RODs is classified.
taxonRank	Character. The taxonomic rank of the most specific name. Controlled vocabulary: species.
type	Character. Controlled vocabulary: PBR, absence, bibliographic data, or error.
basisOfRecords(*)	Character. The specific nature of the data record. Controlled vocabulary: MaterialCitation.
locality(*)	Character. Municipality where the occurrence data was recorded.
decimalLatitude(*)	Numeric (double). The geographic latitude (in decimal degrees, using the spatial reference system given in geodeticDatum) of an occurrence record.
decimalLongitude(*)	Numeric (double). The geographic longitude (in decimal degrees, using the spatial reference system given in geodeticDatum) of an occurrence record.
coordinateUncertaintyInMeters(*)	Numeric (integer). The horizontal distance (in metres) from the given coordinates describing the radius of the smallest circle encompassing the whole study area.
geodeticDatum(*)	Character. The ellipsoid, geodetic datum or spatial reference system (SRS) upon which the geographic coordinates given in decimalLatitude and decimalLongitude are based.
verbatimEventDate(*)	Character. The period in which the EA fieldwork was conducted.
eventDate	Character. The date-time or interval during the occurrence data was generated. It is based on the verbatimEventDate or the ROD publication year.
CEEAcategory	Character. The species threat category on the CEEA or LESRPE. Controlled vocabulary: LESRPE, VU (vulnerable) or EN (endangered).
bibliographicSource	Character. The bibliographic source, institution or others providing the occurrence data indicated on the ROD, if applied. Other related information. Free text.
individualCount	Number (integer). The number of organisms indicated on the ROD, if applied.
verbatimIdentification	Character. The scientific, common name or synonym of the species found in the ROD.
duplicate	Binary. Controlled vocabulary: yes or no.

## Additional information

There were 4,049 documents published in the BOE from 1994 to February 2023 and 3,202 of them are within the “Other provisions” database, where RODs are found. Species listed in the CEEA or the LESRPE were cited in 1,256 (39.2%) RODs. Of the total pronouncements reviewed citing targeted species, only 18.5% (232 out of 1,256) documents contained PBRs.

According to the International Union for Conservation of Nature (IUCN 2025), the conservation status of the 59 species with PBRs in RODs ranges from "Least Concern" (LC) to "Critically Endangered" (CR). A total of 18 species are listed in a threatened category (1 CR, 3 "Endangered" EN and 14 "Vulnerable" VU) at the European level. The remaining 41 are listed in "Near Threatened" NT (18) or LC (33), although three of them are threatened at the Mediterranean level (2 VU and 1 EN) and one at the global level (VU).

## Supplementary Material

3F0202DC-9E25-57D9-B343-06EA169A73EC 10.3897/BDJ.13.e142302.suppl1 Supplementary material 1 Species records recovered from RODs Data type Occurrences Brief description Database comprising all the species records recovered from RODs, including Primary Biodiversity Records (PBRs), literature-based citations and absences. File: oo_1213587.txt https://binary.pensoft.net/file/1213587 Telletxea, M.
